# Flexural Behavior of HPFRCC Members with Inhomogeneous Material Properties

**DOI:** 10.3390/ma8041934

**Published:** 2015-04-21

**Authors:** Kyung-Joon Shin, Kyu-Hyeon Jang, Young-Cheol Choi, Seong-Cheol Lee

**Affiliations:** 1 Department of Civil Engineering, Chungnam National University, 99 Daehak-ro, Yuseong-gu, Daejeon 305-764, Korea; E-Mail: kjshin@cnu.ac.kr; 2 Construction Division, Posco Engineering, Incheon 406-840, Korea; E-Mail: khjang727@poscoen.com; 3 High-Tech Construction Materials Center, Korea Conformity Laboratories, Seoul 153-803, Korea; E-Mail: zerofe@kcl.re.kr; 4 Department of NPP Engineering, KEPCO International Nuclear Graduate School, 658-91 Haemaji-ro, Seosaeng-myeon, Uljugun, Ulsan 689-882, Korea

**Keywords:** fiber reinforced concrete, HPFRCC, beam, crack width, inhomogeneous, Monte Carlo simulation

## Abstract

In this paper, the flexural behavior of High-performance Fiber-Reinforced Cementitious Composite (HPFRCC) has been investigated, especially focusing on the localization of cracks, which significantly governs the flexural behavior of HPFRCC members. From four points bending tests with HPFRCC members, it was observed that almost evenly distributed cracks formed gradually, followed by a localized crack that determined the failure of the members. In order to investigate the effect of a localized crack on the flexural behavior of HPFRCC members, an analytical procedure has been developed with the consideration of intrinsic inhomogeneous material properties of HPFRCC such as cracking and ultimate tensile strengths. From the comparison, while the predictions with homogeneous material properties overestimated flexural strength and ductility of HPFRCC members, it was found that the analysis results considering localization effect with inhomogeneous material properties showed good agreement with the test results, not only the flexural strength and ductility but also the crack widths. The test results and the developed analysis procedure presented in this paper can be usefully applied for the prediction of flexural behaviors of HPFRCC members by considering the effect of localized cracking behavior.

## 1. Introduction

Cement-based materials such as mortar and concrete have long been used as one of the major construction materials. However, these materials are characterized by a relatively low tensile strength, accompanied by a brittle failure. Among the various methods developed to overcome such weaknesses, the application of fibers exhibiting high resistance to tension enhances the tensile performance of cement-based materials [1,2].

Fiber reinforcements are known for improving the toughness of fiber-reinforced concrete (FRC) since the crack bridging effect will better distribute cracks and reduce crack width. However, controlling the cracking behavior with conventional macro fibers is difficult since these fibers do not engage until the concrete or mortar matrix has cracked.

Recently, several researchers have developed a high-performance fiber-reinforced cementitious composite (HPFRCC) [3,4,5,6], and they have shown that the HPFRCC can provide a possible solution for serious localized wide cracks, since it can induce a number of finely distributed cracks. In addition to this advantage, the salient feature of the HPFRCC lies in its strain hardening behavior with large deformation capacity, which conventional fiber-reinforced concrete cannot easily obtain. These multi-cracking and strain-hardening characteristics are achieved by many distributed cracks formed sequentially before a HPFRCC member failed after one of the cracks is localized. Since the tensile stress–crack width relationship of HPFRCC is much different from that of conventional FRC, many studies have been conducted to investigate and predict the behavior of HPFRCC, especially for tensile and flexural responses.

During recent years, the stress–crack width relationship of concrete and HPFRCC has been widely investigated. The pseudo-strain hardening properties of Engineered Cementitious Composite (ECC) under tension are often described as a bilinear stress–strain relation, since this assumption accurately describes the important characteristics of this material behavior. Maalej and Li [7] and JSCE TC 334 [8] use an elastic–plastic relation, which is a function of strain, without considering cracks. However, in order to describe the failure of HPFRCC, crack localizations need to be considered. Ostergaard *et al.* [9] describe the localizing process of ECC using a bilinear softening relation, using the function of crack width to incorporate the influence of the localization in the flexural analysis. However, it should be noted that, in their model, a crack starts to occur when the crack is localized. Therefore the model cannot describe the initial crack width properly, although this can be ignored because the ECC material shows only distributed fine micro-cracks before the localization.

For the flexural behavior of the HPFRCC beam, several methods have been proposed based on the geometry of a beam. For a notched flexural beam, a fracture-based method has usually been used. Maalej and Li [7] have estimated the flexural strength of fiber cementitious composites, and several researchers have predicted the flexural behavior of FRC [10] and ECC [11] using a fracture-based model. For an un-notched beam, a hinge model or section analysis method has commonly been used. Ostergaard *et al.* [9] adapted a hinge model that includes tensile strain hardening and tension softening to predict the flexural behavior of the ECC. A section analysis method was also used for the analysis of convectional FRC [12] and ECC [13]. Recently, a meso-mechanical approach [14] has been developed to explicitly simulate the contribution of matrix and fibers and their mutual interaction. This approach was adopted by Caggiano *et al.* [15] to simulate the flexural behavior of FRC beams with a notch through being implemented in a cracked-hinge model [16].

Among many models, the analytical methods that consider the average cracks over the beams are effective for the investigation of the average tensile and flexural behavior of HPFRCC. In reality, the cracks of HPFRCC form and widen as the applied deformation (or load) increases and one of these cracks localizes. However, simple adaption of homogenous material properties can result in an overestimation in ductility, and maximum crack width can be underestimated, since failure is usually accompanied with a localized crack. The strength of the matrix at the location of this localized crack is generally lower than the strength in other regions of the specimens.

Consequently, the development of an analysis model that considers the inhomogeneous multiple cracking behavior in HPFRCC members is needed. In order to achieve this goal, this study conducts an experimental program for the flexural behavior of the HPFRCC member. The flexural analysis model with a layered section method has also been presented and compared with the test results.

## 2. Experimental Study

### 2.1. Test Outline

Several tests were conducted to measure the mechanical properties of HPFRCC while a typical HPFRCC mixture was employed to induce multiple cracks. Based on several previous studies [12,17,18], the mixture proportion was determined. Fly-ash was used to improve the fiber–matrix interface characteristics based on the previous results of Li *et al.* [18] and Kim *et al.* [19,20].

### 2.2. Materials and Mix Design

The mix design of HPFRCC differs significantly from that of conventional concrete. HPFRCC mix compositions are characterized by the use of micro fibers and silica sand with a small diameter, and the exclusion of coarse aggregate. The water/cement (W/C) ratio was 0.55, the water/binder ratio was 0.34, and the sand/cement (S/C) ratio was 0.6 by weight. Ordinary portland cement and fly-ash were used in the mixture, and a small amount of super plasticizer (0.5% of cement weight) was added. To achieve the multiple cracking behavior, Polyvinyl alcohol (PVA) fibers of 0.04 mm in diameter and 8 mm in length were added to the mortar matrix at a 2% fiber/volume ratio. The tensile strength of the fiber, provided by a manufacturer, was about 1.6 GPa. Details of the mixture design have been presented in Table 1.

**Table 1:** High-performance fiber-reinforced cementitious composite (HPFRCC) mixture design.

W/C	S/C	Unit Content (kg/m^3^)				8 mm PVA Fiber
		C	W	S	Fly Ash	
0.55	0.6	727	400	436	436	2%

### 2.3. Compressive Behavior

**Figure 1:**
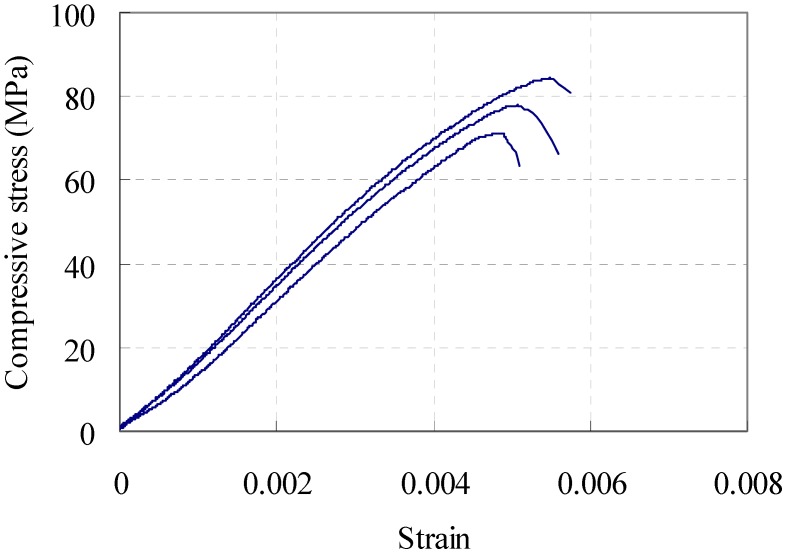
Stress–strain behavior of high-performance fiber-reinforced cementitious composite (HPFRCC) in uniaxial compression.

Cylinder specimens of 100 mm in diameter and 200 mm in height were made in order to measure the compressive strength and elastic modulus. The top and bottom surfaces of each cylinder were first ground. A displacement control method was used to apply the load and the complete load–deformation data were measured using a compressometer. Figure 1 shows the measured compressive behavior. The test was stopped before the specimen completely lost its resistance due to the limitations of the measuring apparatus. It is noted that the specimens exhibited significant residual compressive stress even after reaching the compressive strength due to the bridging effect of embedded fibers.

### 2.4. Tensile Behavior

**Figure 2:**
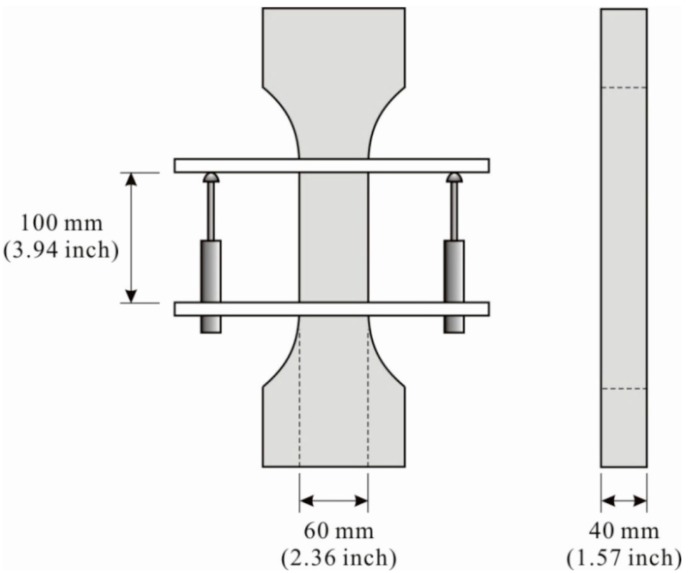
Specimen geometry and test setup for the direct tension test.

**Figure 3:**
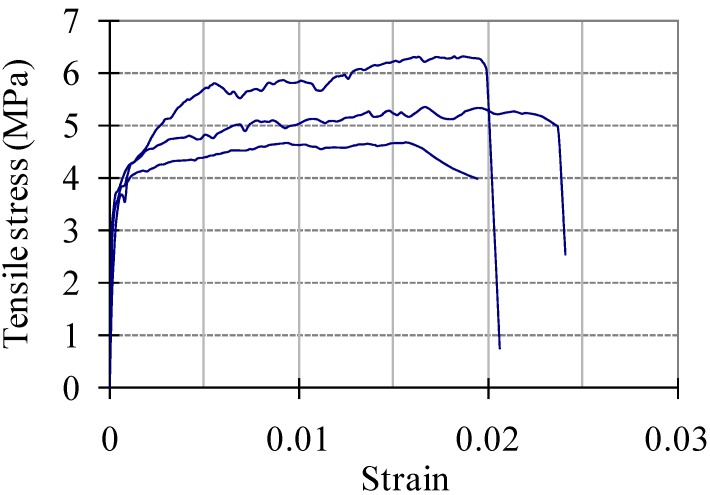
Stress–strain behavior of HPFRCC in uniaxial tension.

A series of direct tensile tests were performed to measure the tensile stress–strain relation. Tests were conducted using an MTS 810 machine (MTS Systems Cooperation, Eden Prairie, MN, USA) with a loading rate of 0.2 mm/min. Two electric displacement transducers were attached to both sides of the center of the tensile specimen in order to monitor the elongation. To avoid fractures outside the measurement area, both ends of the specimens were made in the shape of a dog-bone. In addition to the tensile stress–strain curves, the ultimate tensile strength was measured, as was the ultimate tensile strain. Figure 2 shows the specimen geometry and the test setup. The measured stress–strain curves are presented in Figure 3. The measured average ultimate strength and ultimate strain are 5.45 MPa and 0.0193, respectively.

### 2.5. Flexural Behavior

Flexural tests were conducted to observe the flexural and cracking behaviors of HPFRCC including load–deflection and load–CMOD (Crack Mouth Opening Displacement) relationships. The beam specimens of 100 mm × 100 mm × 400 mm (width by height by length) were made. After casting, they were stored at room temperature for 1 day prior to demolding, then cured in water until tested at the age of 28 days.

**Figure 4:**
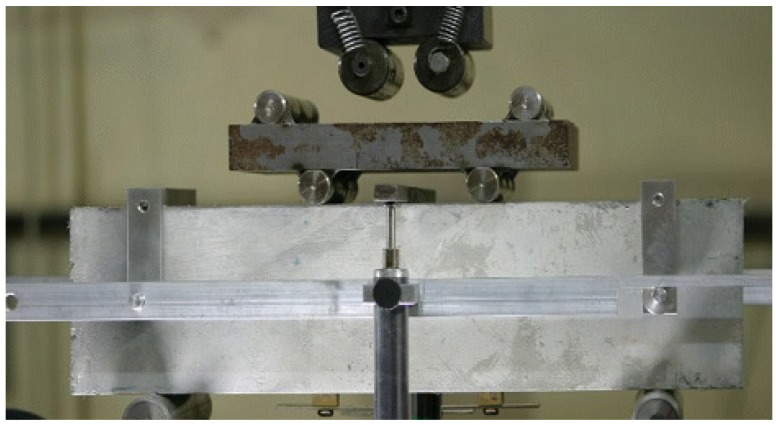
Test setup for four-point flexural test.

**Figure 5:**
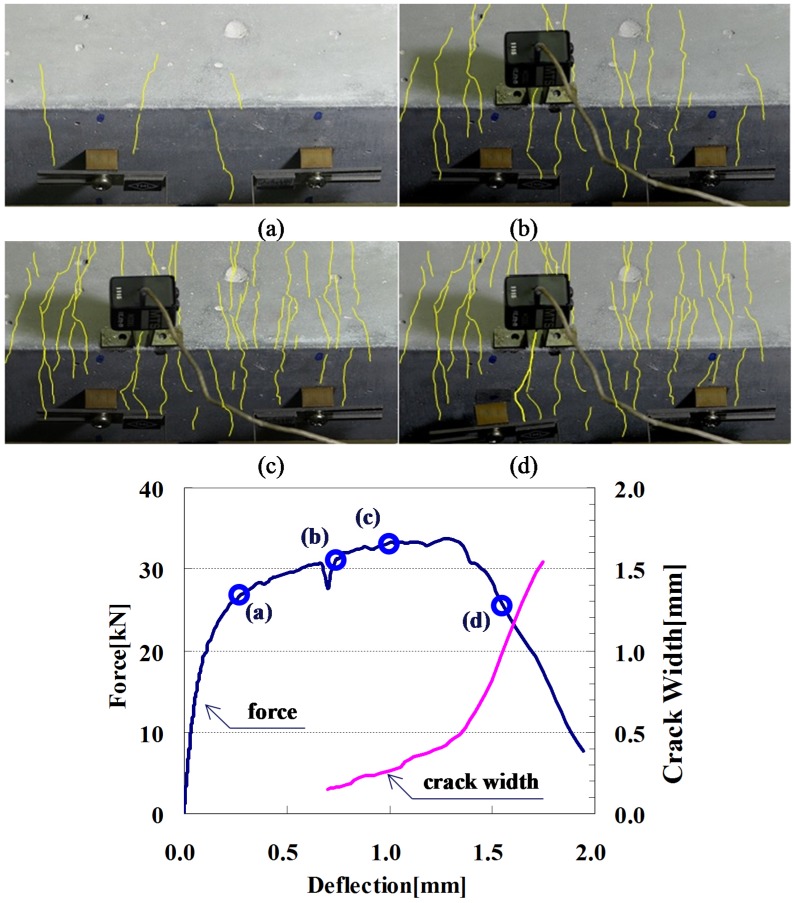
Load–deflection relationship and cracking behavior of a HPFRCC beam. (**a**) initial cracking stage; (**b**) crack gauge attached; (**c**) distributed fine cracks; (**d**) localized crack.

Figure 4 shows the setup of the flexural test on HPFRCC beam in a four-point bending with a pure span of 300 mm, which is the length between the supports. Relative displacements between the supports and the center point were measured at the center of the beam through the instrument attached on the beam, as shown in Figure 4. The tests were conducted in a hydraulic servo-controlled testing machine with a closed-loop system. The crack-width was measured using a crack mouth opening displacement (CMOD) gauge. When a crack was detected, the actuator displacement was stopped and the initial crack width was measured using a microscope. Then, the crack width was monitored with a CMOD gauge, as shown in Figure 5.

Figure 5 shows the cracking process of a typical specimen with its load–deflection curve. Initially, with the first crack, the stiffness on the load–deflection curve decreased. In Figure 5a, a few fine cracks, which were hard to observe with a naked eye, are shown. As the deflection increased from Figure 5a to Figure 5b, more cracks gradually formed, but the crack widths still remained fine without localization. In Figure 5b, several cracks were observed, and a CMOD gauge was attached at the widest crack in order to trace the crack width. The initial crack width was measured using a microscope. From Figure 5b to Figure 5c, the number of cracks did not increase, but the deflection increased continuously by evenly distributing additional deformation into every crack. Therefore, the crack widths proportionally increased without localization until the load reached its maximum. Finally, one of the cracks was localized, as shown in Figure 5d. It should be noted that the crack measured by the CMOD gauge was the widest among the observed cracks for this specimen, and the beam failed at the section with the largest crack.

## 3. Analysis Model for Cracking and Flexural Behavior of HPFRCC Beams

Since cracking occurs progressively over the region of tension as the load (or deflection) increases, the inhomogeneous tensile behavior needs to be considered in order to reasonably simulate and predict the flexural and cracking behavior of the HPFRCC. A homogeneous analysis approach cannot describe the crack localization process so that the flexural ductility is overestimated. Therefore, an inhomogeneous analysis approach has been applied in this study.

### 3.1. A Sequential Cracking Behavior Model

**Figure 6:**
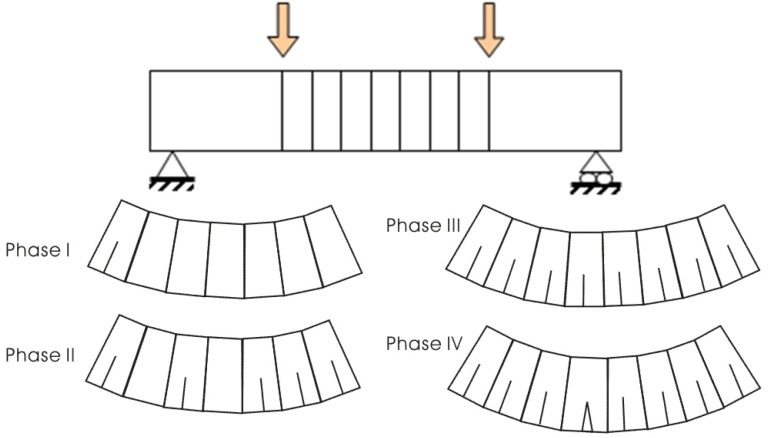
Model for pure bending region in flexural member.

Unlike conventional FRC members in which an initial crack is mostly dominant, HPFRCC shows a pseudo-strain hardening behavior with finely distributed multiple cracks. This multiple cracking process occurs in sequence as the applied tensile load increases, as illustrated in Figure 6. After tensile stress reaches the maximum affordable tensile stress of the weakest section, the crack at the weakest section is localized. Therefore, through these sequential cracking processes, each crack shows a different crack opening history due to inhomogeneous material properties.

In order to simulate this cracking behavior caused by inhomogeneous material properties, the pure bending region of the specimen was assumed to consist of several segments, and each segment was assigned to different material properties. These consisted of first cracking strength and ultimate strength which were randomly assigned according to the defined probability distributions, based on the proposal in JSCE TC 334 [8]. The detailed information for the distributions is provided for a problem in the later Section 4.2.

### 3.2. A Layered Section Analysis Method

A layered section analysis method, which has been widely used for the analysis of flexural behavior of a concrete beam [21,22,23], has been adapted to describe the deformation and crack width of a HPFRCC beam.

**Figure 7:**
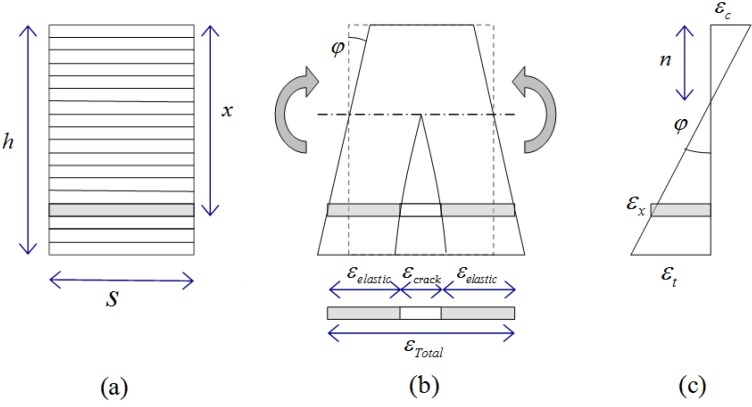
Layered model subjected to flexure: (**a**) un-deformed shape; (**b**) deformed shape; and (**c**) strain distribution over the height.

In the section analysis model without axial stress, the deformation of a segment can be described by the curvature (φ) and the depth of the neutral axis (*n*). Based on Bernoulli’s principle that the longitudinal strain through the height in a beam is proportional to the distance from the neural axis [21], the following relationship is easily derived:

(1) $$\text{φ}=\frac{{\text{ε}}_{c}}{n}=\frac{{\text{ε}}_{c}+{\text{ε}}_{t}}{h}$$

where *h* is the height of the beam, ε*_c_* is the strain at the top fiber, and ε*_t_* is the strain at the bottom fiber. Hence, the strain (ε*_x_*) at a specific depth (*x*) can be calculated by using Equation (2), as shown in Figure 7:

(2) $${\text{ε}}_{x}=\text{φ}\cdot x-{\text{ε}}_{c}$$

The depth of the neutral axis can be found from the force equilibrium at the cross section as the sum of the longitudinal forces at each layer should be equal to the external axial force. In this research, a false position method [24] was used to find the nonlinear solutions.

### 3.3. Constitutive Models for the Materials

**Figure 8:**
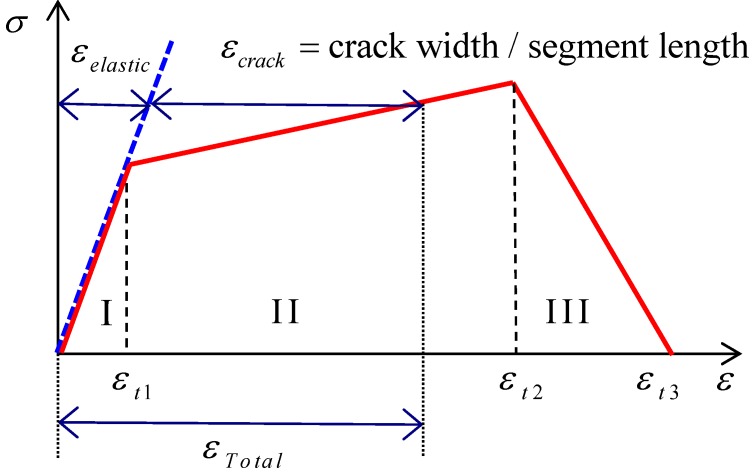
Total tensile stress–strain relationship in uniaxial tension.

HPFRCC beams have been modeled with several segments, and the length of each segment is assumed to be equal to the final crack spacing, so that each segment consists of a single crack and elastic composite parts, as shown in Figure 7b. The crack is assumed to occur when the tensile stress exceeds the tensile strength of the matrix. The elastic part behaves like an elastic material while the crack is activated after the tensile crack occurs. Therefore, when the stress is below the tensile strength of the matrix, the crack width is zero and the segment behaves as an elastic one. After the tensile stress exceeds the tensile strength of the matrix, the total strain of the segment is assumed to follow the relationship shown in Figure 8 while the elastic part still follows the elastic behavior. The total tensile strain of a segment can be calculated from the sum of the strains for the elastic parts and crack width, as illustrated in Figure 8. In Figure 8, Region I represents the elastic state before a first cracking. Region II represents the status when the crack is activated but not localized. In Region II, both the elastic parts and the crack can resist an increased load, which reflects the fiber-bridging and strain-hardening behaviors of HPFRCC in tension. The localization process is described in Region III. For the segment with a localized crack, with an increase in total tensile strain, the tensile stress decreases and the tensile strain of the elastic parts decreases while the width of a localized crack increases. The crack strain in the segment can be calculated by subtracting the elastic strain from the total strain, so the crack width is calculated by multiplying the length of the segment:

(3) $${\text{δ}}_{\text{crack}}=s\cdot ({\text{ε}}_{\text{tot}}-{\text{ε}}_{\text{elastic}})$$

Thus, the present model can include the influence of the tensile stress-crack width relationship from the moment of micro crack initiation to the localization. For the apparent total tensile stress–strain relationship of HPFRCC, the multi-linear model shown in Figure 8 has been used in this paper. In the model, the following values are estimated from the test results shown in Figure 3: ε_*t*1_ = 0.0004; ε_*t*2_ = 0.0193; ε_*t*3_ = 0.0212.

When dealing with compressive behavior, a parabolic equation was used, as is commonly done with plain concrete [25] and with a conventional FRC [12,26,27]. On the other hand, although JSCE TC 334 [8] proposed a parabolic equation without a descending region, a linear relation has been also commonly used to describe the compressive behavior of HPFRCC. Several researchers [7,28,29] have proposed a bi-linear equation for tensile strain hardening in fiber-reinforced composites such as ECC.

From the compressive test results shown in Figure 1, the constitutive model for the compressive behavior is expressed as:

(4a) $$f_{c}={f_{c}}^{\prime }({\text{ε}}_{c}/{\text{ε}}_{0})\;\;\;\text{for}\;\;\;{\text{ε}}_{c}\leq {\text{ε}}_{0}$$

(4b) $$f_{c}={f_{c}}^{\prime }(1-0.5\frac{{\text{ε}}_{c}-{\text{ε}}_{0}}{{\text{ε}}_{f}-{\text{ε}}_{0}})\;\;\;\text{for}\;\;\;{\text{ε}}_{c}>{\text{ε}}_{0}$$

where *f_c_* is the concrete stress at strain ε*_c_*, ${f_{c}}^{\prime }$ is the maximum compressive strength at strain ε_0_, and ε*_f_* is the strain at the compression failure. All values are positive for the compression. The test results show the average compressive strength and the corresponding strain was measured at 76.28 MPa and 0.005, respectively.

### 3.4. Determination of Segment Length

In order to calculate crack width from tensile strain in a segment, the length of the segment, *s*, should be carefully selected. Ulfjaer *et al.* [30] reported that the best agreement with the results from a numerical model was obtained when the segment length of conventional concrete beams was one half the height of the beam (*s* = 0.5 *h*). In addition, the same ratio for the segment length was successfully applied to fiber-reinforced concrete [16]. Different from conventional normal or fiber-reinforced concrete, which usually shows a single crack in a small specimen, several cracks occurred in a flexural specimen with HPFRCC. In this study, it is assumed that each segment has one crack so as to simulate every crack in the specimen individually. The length of a segment is assumed to be the average crack spacing at the failure, and this spacing was measured in the experiment. For the specimens of 100 mm × 100 mm × 400 mm, the length of the segment has been assumed to be 14.29 mm, since the number of cracks was about 7 (10, 5, and 6 cracks for the 3 specimens).

### 3.5. Calculation of Deflection

**Figure 9:**
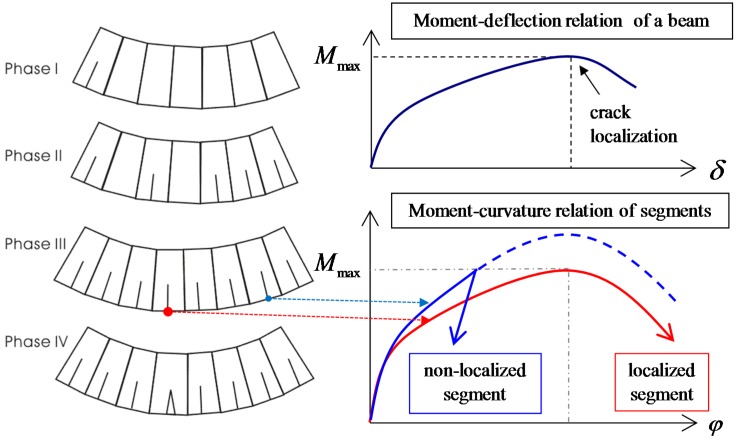
Deflection of a beam and curvature of segments.

Figure 9 shows that while the flexural behavior of a HPFRCC beam follows the apparent moment–deflection curve, two representative behaviors can be observed in the segments; the weakest segment with a localized crack follows the descending part in the moment–curvature relationship, and the others follow the unloading path without localization when the applied moment reaches the maximum moment, which is less than the maximum capacity of those segments. In other words, the crack in the weakest segment continuously opens up to the failure, while the other cracks close after the beam reaches its maximum capacity.

To calculate the deflection of the beam, therefore, the segments need to be classified into the localized and non-localized elements, as mentioned above, because the localized element exhibits ductile softening behavior while the non-localized elements follow the unloading path after the beam reaches the maximum moment, as illustrated in Figure 9. Deflection induced by non-localized segments can be calculated according to the elastic theory [31] using Equation (5):

(5) $${\text{δ}}_{\text{nonlocal}}=\int {\text{φ}}_{\text{nonlocal}}x\text{d}x$$

**Figure 10:**
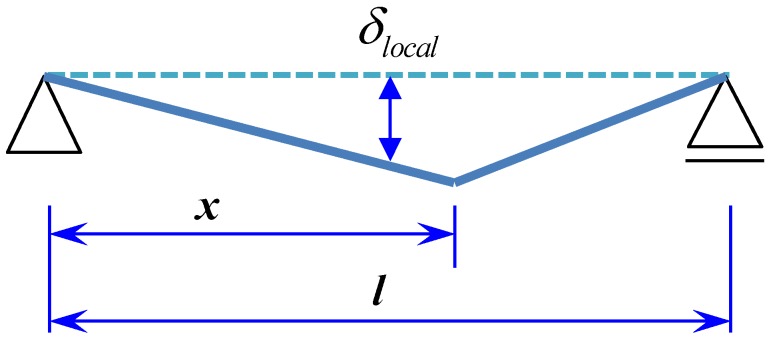
Deformation of beam in rigid-body rotation.

For the localized element, the elastic theory can be applied only before the crack localization. After the localization, the calculation of the deflection caused by the segment with a localized crack can be based on a rigid-body rotation theory and a plastic deformation concept, as shown in Figure 10. The deflection induced by the localization can be calculated from the following equation:

(6) $${\text{δ}}_{\text{local}}=\int \frac{l-x}{2}{\text{φ}}_{\text{local}}\text{d}x$$

Consequently, the deflection of the specimen can be calculated through the superposition of the elastic and plastic deformations, since the deflection can be calculated through integrating curvatures along the beam:

(7) $${\text{δ}}_{\text{total}}={\text{δ}}_{\text{nonlocal}}+{\text{δ}}_{\text{local}}$$

## 4. Analysis Results

### 4.1. Flexural Deformation Behavior

**Figure 11:**
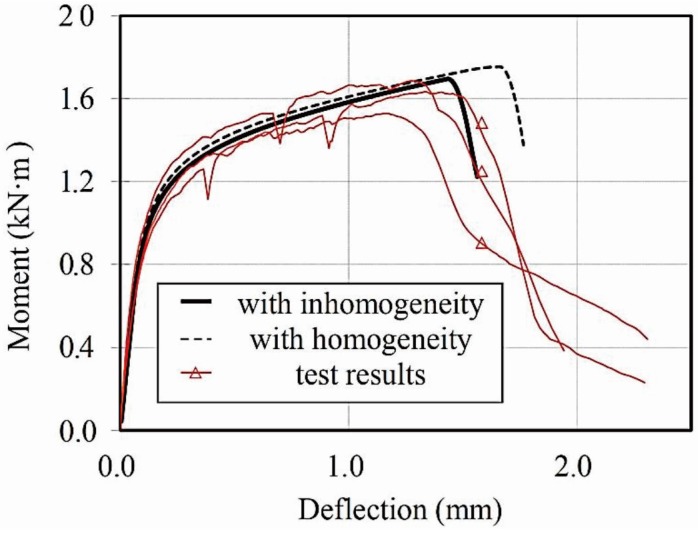
Moment–deflection curve of specimen.

The load–deflection curves of the beams tested in this study have been compared in Figure 11 with the analysis results that are predicted using homogeneous or inhomogeneous material properties. Unlike the analysis result using a homogeneous condition, the proposed model can produce many varying results depending on the material parameters selected for the simulation. An example of one of these results is shown in Figure 11.

As shown in the figure, the effect of localization influences the flexural ductility significantly. When the material properties were assumed to be homogeneous, the analysis overestimated the flexural ductility of the beam, since the effect of the localized crack, that is loading and unloading elements, is not included in the analysis. On the other hand, the proposed model considering inhomogeneous material properties showed better agreement with the test results.

For realistic simulation of flexural behavior of HPFRCC, the localized and non-localized behaviors need to be considered, because HPFRCCs have multiple cracking and deformation hardening as characteristic behaviors; in addition, these multiple cracks do not behave the same when crack bridging [9,32]. Non-uniform distribution of fibers can also be another cause for variation in the crack-bridging behavior [33,34].

**Figure 12:**
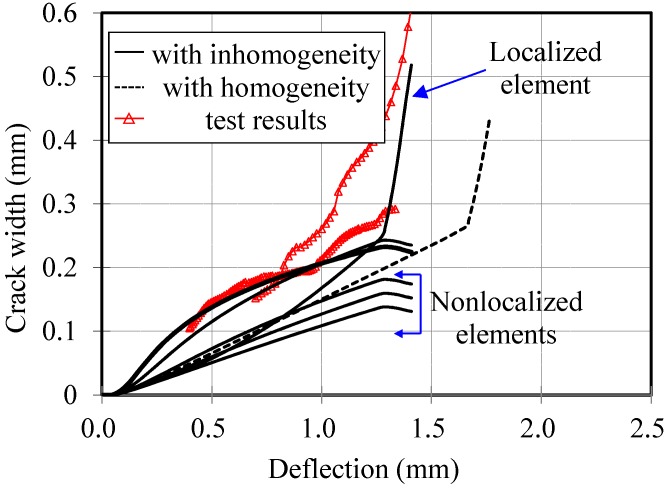
CMOD–deflection curve.

The proposed model better predicts the flexural behavior of HPFRCC beams when the crack widths are compared with each other. The crack widths measured with CMOD gauges in two specimens are compared with the analysis results, as shown in Figure 12. When homogeneous material properties were used in the analysis, every segment and every crack showed identical behavior, so that only one crack width–deflection relationship was obtained. On the other hand, when the inhomogeneous material properties were considered, each segment and each crack showed different histories of the structural behavior. While the crack in the weakest segment opened gradually due to its localization, the crack widths in the other segments increased as the applied load increased, and then decreased due to unloading. Consequently, the analysis with homogeneous material property gives only one crack width–deflection response, shown by the dotted line in Figure 12, while the analysis with inhomogeneous material properties gives several crack width–deflection responses; one response is for a localized element, while the other response is for non-localized elements. Through the comparisons presented in Figure 12, which give the crack widths predicted by each model, it can be proved that the proposed model can describe the maximum crack width of HPFRCC member under flexural loading reasonably well. Therefore, it can be concluded that the multiple cracking behavior of HPFRCC members under flexural loading can be reasonably described by the proposed model.

### 4.2. Monte Carlo Simulation of Flexural Behavior

The previous section has shown that the proposed method, which considered inhomogeneous material properties of each segment, accurately described the flexural behavior of HPFRCC beams including flexural ductility as well as crack width. With the proposed method, it should be noted that the analysis results can be variable, since different material properties are assigned for each analysis. In this study, therefore, the Monte Carlo simulation [35], which is widely used in an analysis with random variables, has been implemented in the proposed method in order to consider the uncertainty for the material properties of HPFRCC. The simulation analysis was conducted 1000 times, and results with 99% certainty were extracted.

Normal distributions were assumed for the test parameters, since no sufficient data have been accumulated so far for this research area. The first cracking strain and strength, and the ultimate strain and strength were randomly assigned according to the probability distributions assumed. Measured results are used for the mean values of the normal distributions. However, the coefficients of variation were adapted from the Kanakubo’s round-robin test [36] since the number of measurements was limited. The coefficients of variation (CoV) for the first cracking strain and strength were 16.5% and 22.5%, respectively. The CoVs for the ultimate cracking strain and strength were 24.3% and 9.4%.

**Figure 13:**
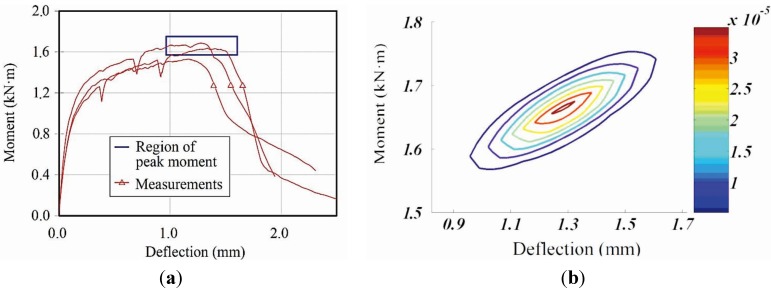
Region of peak moment and deflection obtained with 99% certainty: (**a**) peak moment and deflection region; (**b**) probabilistic distribution on peak moment and deflection.

The result obtained from the simulation is compared with the test results in Table 2 and Figure 13. The maximum moment was 1661 kN·mm with a standard deviation 46.06. The average deflection at the maximum moment was 1.284 mm and the standard deviation was 0.164. The measured deflections are within an interval of 99% confidence, between 1.014 and 1.554 mm.

**Table 2:** Analysis and test results at a maximum moment.

Classifications		Moment (kN·m)	Deflection (mm)	Crack Width (mm)
Results with the homogeneity		1.754	1.662	0.266
Monte Carlo simulation	Average	1.661	1.284	0.203
	Standard Deviation	0.046	0.164	0.010
Test results	Specimen 1	1.529	1.176	0.258
	Specimen 2	1.687	1.273	0.421
	Specimen 3	1.635	1.337	not available
	Average	1.617	1.262	0.340

For maximum crack widths, the measurements were greater than the predictions. This result may have been caused by the limitations of the prediction model and measuring method. The crack widths were measured using a CMOD gauge, which measures the change of length between two steel tips as shown in Figure 5, so that it includes deformations caused by the section other than the targeting crack width itself. For HPFRCC, Li *et al.* [17,18] have reported that many invisible cracks form in the matrix. Therefore, the measured crack width tends to be overestimated. Meanwhile, since the elastic part of the material model adapted in this study is assumed to be elastic after the crack forms, predictions tend to underestimate the crack width.

## 5. Conclusions

This study carried out a combined experimental and theoretical investigation of the flexural behavior of a HPFRCC. A numerical approach for modeling the flexural and cracking behaviors of HPFRCC was proposed:

1. Compressive and tensile constitutive relations were proposed based on the experimental results. A bi-linear relation was assumed for the compression. Regarding the tensile behavior, the total strain was assumed to be a sum of the elastic and crack strains.
2. In order to develop a realistic model that could account for sequential multiple cracking and localization behavior of the HPFRCC, the pure bending region was divided into several segments with random material properties. The properties of the segments are assigned based on the probabilistic distribution of the material parameters.
3. A Monte Carlo simulation had been conducted in order to consider the uncertainty for the material properties of HPFRCC. Results of the Monte Carlo simulation showed that the test results are within the region of prediction with 99% certainty.

With this proposed method, the complete flexural behavior of the HPFRCC beam, such as load–deflection and load–crack width responses including localization of cracks, could be rationally predicted.
